# Polymyalgia rheumatica and large vessel vasculitis: a case report

**DOI:** 10.1093/rap/rkaa049

**Published:** 2020-10-20

**Authors:** Michael Dare, Bhaskar Dasgupta, Anupama Nandagudi, Krisztina Szabo-Kocsis

**Affiliations:** r1 Rheumatology Connect Health Community Rheumatology Service, East Kent, Kent; r2 Rheumatology, Mid & South Essex University Hospitals NHS Foundation Trust, Basildon, Essex; r3 Rheumatology Service Connect Health Community Rheumatology Service, South West Essex, UK

**Key Message**

- Large vessel vasculitis may present initially as a mechanical presentation and develop over time.

Dear Editor, PMR is a common inflammatory disease found in elderly patients and results in a high burden of long-term CS-based therapies [1]. The disease usually presents with an abrupt onset of primarily shoulder girdle and hip girdle pain and stiffness and is found exclusively in cohorts >50 years of age. Constitutional symptoms of fatigue, weight loss, pyrexia and peripheral synovitis have also been reported. More importantly, PMR is associated with GCA in 10–20% of cases [1]. Currently, GCA is subdivided into cranial disease (headache, jaw pain, visual and ischaemic symptoms) *vs* extracranial large vessel disease (constitutional symptoms, limb claudication, poor CS response, aortic and other large vessel involvement on imaging). Therefore, PMR is thought to belong to a disease spectrum that includes GCA, large vessel vasculitis and PMR [2].

In addition, other conditions, such as neoplasia, bacterial endocarditis, osteomyelitis and other inflammatory conditions, can masquerade as PMR, which can make it difficult to ascertain the diagnosis [3].

We present a case of PMR with associated large vessel vasculitis. This supports a new concept of an overlap between PMR and large vessel vasculitis as part of a wider GCA spectrum. Normally, such a complex diagnosis is made in a secondary care setting within a rheumatology clinic. Of particular importance in the management of this case is that the diagnosis of large vessel GCA manifesting as PMR was first queried by a physiotherapist with an interest in rheumatology.

## Case report

A 72-year-old Caucasian woman was referred to a community rheumatology service with insidious onset of lower back pain and stiffness. The symptoms gradually progressed to involve the shoulder girdle, with prominent systemic symptoms of weight loss, fever and malaise. There were no symptoms suggestive of CTD or spondyloarthritis. Owing to severe headache, the patient was seen several times in primary care, in Accident and Emergency, and was found to have a normal CT brain.

The patient’s shoulder and hip girdle symptoms had largely resolved in the 3 weeks before consultation without any intervention, after which she gradually developed severe right unilateral frontal and temporal headaches in additiont o right upper limb claudication.

On clinical examination, there was no peripheral arthritis. Shoulder active range of motion was reduced. The chest was clear on auscultation, and there was no lymphadenopathy. There was no temporal or axillary tenderness. The left temporal pulse was palpable, whereas the right was absent. The peripheral radial and ulnar pulses were normal.

Laboratory investigations revealed a CRP of 16 mg/l, ESR 17 mm/h, normal full blood count, normal renal and liver function and a mild thrombocytosis. A previous lumbar spine radiograph and CT brain were normal.

Taking into account the history and clinical presentation, a suspected diagnosis of polymyalgic onset large vessel GCA was made, and the patient was referred to the Acute Medical Unit. This is a secondary care-based service for acutely unwell patients referred from primary care. She was started on high-dose oral prednisolone 60 mg daily, with rapid improvement in her symptoms.

**Fig. 1:**
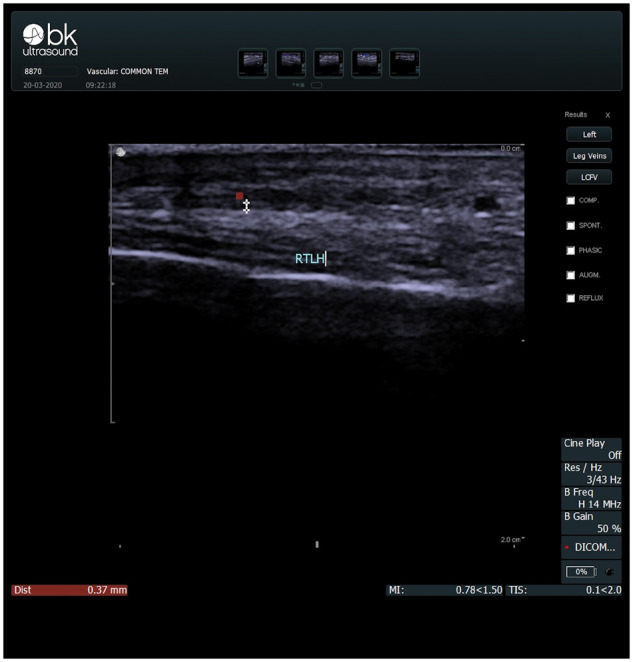
Right temporal artery

Before initiation of CSs, as mentioned previously, this patient had been investigated extensively with numerous blood tests, CT brain, lumbar spine radiograph and chest radiograph. Subsequently, a temporal US was found to be positive for temporal arteritis, demonstrating a halo and intima–media thickness for the right temporal artery of 0.37 mm, as shown in Fig. 1.

Although the initial presentation was not unusual for PMR, several important points relating to PMR and large vessel vasculitis can be appreciated in this case. It is noteworthy that not every onset of PMR involves the proximal shoulder girdle. According to Kniazkova *et al.*[*3*], 15–30% of patients can present with lower hip girdle pain and stiffness at onset; however, true PMR will progress to involve the shoulders [4]. This is of particular importance, because we should not exclude PMR if the shoulders are not involved at the initial presentation.

GCA has been reported in 10–20% of PMR patients, and these conditions have a large overlap. According to Dejaco *et al.* (2017) [2], previous focus was placed on cranial vasculitis, but recent research has suggested a strong overlap of cranial vasculitis and large vessel extracranial vasculitis with PMR. This form of large vessel GCA can be present in ≤30% patients with PMR, particularly those who do not respond adequately to low-dose glucocorticoid therapy [2]. This case illustrates a typical example of polymyalgic onset of symptoms, with raised inflammatory markers followed by cranial symptoms (headaches) and thereafter extracranial symptoms (constitutional symptoms, upper limb claudication), and the US imaging confirmed the diagnosis of cranial and large vessel GCA [2].

The attending clinician should always screen for large cranial and extracranial vessel vasculitis as part of the polymyalgic syndrome, especially in patients who present with strong constitutional symptoms. This can require additional tests, such as vascular US or a PET-CT scan for large vessel GCA. Our case report also suggests the need for better training for physiotherapists involved in the assessment/triage of musculoskeletal symptoms of possible rheumatological origin. In this case, the patient initially presented to a Musculoskeletal service Physiotherapist lead as mechanical hip pain. However, after careful history and examination, she was referred to the Acute Medical Unit with suspicion of polymyalgic onset large vessel GCA. We suggest that such formal training should be considered in order that physiotherapists can assist general practitioners in the assessment and management of rheumatological musculoskeletal conditions.

*Funding*: No specific funding was received from any bodies in the public, commercial or not-for-profit sectors to carry out the work described in this manuscript

*Disclosure statement*: The authors have declared no conflicts of interest.
